# Predicting drug adverse effects using a new Gastro-Intestinal Pacemaker Activity Drug Database (GIPADD)

**DOI:** 10.1038/s41598-023-33655-5

**Published:** 2023-04-28

**Authors:** Julia Yuen Hang Liu, John A. Rudd

**Affiliations:** School of Biomedical Sciences, Faculty of Medicine, The Chinese University of Hong Kong, 704, Lo Kwee-Seong Integrated Biomedical Sciences Building, Shatin, New Territories, Hong Kong, SAR People’s Republic of China

**Keywords:** Computational biology and bioinformatics, Gastroenterology, Drug discovery, Drug safety, Drug screening, Target identification

## Abstract

Electrical data could be a new source of big-data for training artificial intelligence (AI) for drug discovery. A Gastro-Intestinal Pacemaker Activity Drug Database (GIPADD) was built using a standardized methodology to test drug effects on electrical gastrointestinal (GI) pacemaker activity. The current report used data obtained from 89 drugs with 4867 datasets to evaluate the potential use of the GIPADD for predicting drug adverse effects (AEs) using a machine-learning (ML) approach and to explore correlations between AEs and GI pacemaker activity. Twenty-four “electrical” features (EFs) were extracted using an automated analytical pipeline from the electrical signals recorded before and after acute drug treatment at three concentrations (or more) on four-types of GI tissues (stomach, duodenum, ileum and colon). Extracted features were normalized and merged with an online side-effect resource (SIDER) database. Sixty-six common AEs were selected. Different algorithms of classification ML models, including Naïve Bayes, discriminant analysis, classification tree, k-nearest neighbors, support vector machine and an ensemble model were tested. Separated tissue models were also tested. Averaging experimental repeats and dose adjustment were performed to refine the prediction results. Random datasets were created for model validation. After model validation, nine AEs classification ML model were constructed with accuracy ranging from 67 to 80%. EF can be further grouped into ‘excitatory’ and ‘inhibitory’ types of AEs. This is the first time drugs are being clustered based on EF. Drugs acting on similar receptors share similar EF profile, indicating potential use of the database to predict drug targets too. GIPADD is a growing database, where prediction accuracy is expected to improve. The current approach provides novel insights on how EF may be used as new source of big-data in health and disease.

## Introduction

Gastrointestinal (GI) motility is highly coordinated by rhythmic electrical signals, called pacemaking activity or slow-waves, controlled by network of interstitial cells of Cajal (ICC). These propagating electrical signals can be easily recorded using a microelectrode array (MEA) platform, and drug-induced effects on GI pacemaking activity can be easily tested using a standardized methodology^1–6^. It is proposed that these electrical signals are the languages representing healthy bowel movement and reflecting non-conscious health and disease conditions of our body, such as, vomiting, diarrhea and constipation.

Electrical data is an enormous type of big-data which is currently highly-underutilized in biology and medicine. To decode the language of rhythmic GI pacemaking signals, we tried to communicate with it through drug treatment to activate or inhibit various receptors, ion channels, enzymes. The final pacemaking signals may integrate signals directly coming from ICC, indirect interactions with enteric neurons and smooth muscles, and signals coming beyond the GI. The current study aims to decode and translate these signals with the help of artificial intelligence (AI) technology, to identify correlations between GI pacemaker activity and health and disease. We collected and integrated a large amount of drug testing data on GI pacemaker activity in our previous publication and unpublished studies, where we used a standardized and robust MEA methodology to test different drugs at different effective doses in different GI segments including, stomach, duodenum, ileum and colon^1–6^. We built a new type of electrophysiological drug database based on changes in 24 signal features extracted from recorded GI pacemaker activity before and after acute drug treatment, which are referred to as “electrical” features (EFs). We named this drug database the Gastro-Intestinal Pacemaker Activity Drug Database, or GIPADD in short. While the GIPADD is continuously growing, this report is created using a cut-off database with 89 drugs and 4867 datasets. The current study aims to explore the potential application of this novel EF drug database, the GIPADD, in predicting drug adverse effects (AEs).

In the newly emerging field of AI drug discovery, AI had been applied in designing drug molecules and predicting drug targets, responses and AEs. Current methodologies mainly used drugs’ physical and chemical properties, AI-predicted or known drug-protein-receptor binding interactions, and genetic or gene expression profile^7, 8^. The current study introduces EF drug profile into this research field. Current available drug AEs prediction AI models have significant limitation in data quality, including bias, non-standardized and non-validated data collection methodologies. Besides, data used mainly came from publications which often present data that supports a hypothesis, instead of a pure factual data presentation, where negative data are very often unreported. Prediction algorithms based on these data suffered significant biased towards what is discovered or hypothesized by human, limiting its ability to discover novel pathways or correlations. Therefore, we propose that creating and maintaining physiological drug databases produced by standardized and validated methodology could be the game-changing solution in AI drug discovery. In which, the GIPADD is produced by a highly-standardized methodology, i.e. drug profiles of every drugs stored in the GIPADD are highly consistent, and therefore, could act as positive and negative controls for each other depending on the testing hypothesis. Besides, GIPADD stores unique EF, which potentially integrates further with other drug databases storing physical, chemical, genetic data. The current study integrates GIPADD with side-effect resource (SIDER) to test the hypothesis to use drug-induced GI pacemaker features to predict drug AEs. Moreover, GI function goes far beyond digestion for absorbing life-supporting nutrients, but contributes to at least 70 percent of our immunity and 95 percent of serotonin release controlling our emotions^9^. It expresses almost all known receptors or proteins that may be found in the brain, liver, reproductive organs and other tissues and organs. Therefore, it is further hypothesized that AEs prediction using GI pacemaker activity could go beyond predicting only GI-related AEs, but also AEs in immunology, cardiovascular, psychology.

## Materials and methods

### Database construction

Datasets accumulated in the database were produced using previously described methodologies^1–6^. In brief, 24 features, including dominant frequency, average frequency, dominant power, amplitude, period, velocity; percentage of contribution in power spectrum divided into percentages of brady-rhythm, normal-rhythm, and tachy-rhythm; signal stability and complexity features including multiscale sample entropy and detrended fluctuation analysis divided into various time window scale; and wave propagation features including change in dominant propagation patterns were automatically extracted using customized and automated analytical programs^4^. Standardized and optimized filters and thresholds settings were included in the analytical programs to remove datasets that did not met baseline signal quality requirements. Datasets used are produced from female *Suncus murinus* aged 1.5–3 months old only. Datasets produced from male animals are not included in the current pilot study.

### Learning datasets construction

All 24 features were normalized into percentage change values by [(X_post-drug_ – X_Baseline_)/X_Baseline_ × 100%] for data with specific units, and (%X_post-drug_ – %X_Baseline_) for features which were originally presented in percentage of contribution. Normalized features and the drug name, tested dose and tissue-type were further merged with the online side effect resource (SIDER) database (version downloaded in Oct 2021)^10^ based on matched drug names. Sixty-six common AEs were selected for this study. Drug was marked as ‘1’ indicating a positive correlation with the AEs. If the drug was clinically used as an indication of the listed AEs, a negative correlation ‘0’ was marked regardless of the positive correlation to the AEs. This step was to minimize the potential false reports of AEs due to existing conditions in patients. Otherwise, a negative correlation ‘0’ was marked for all drugs without the listed side effect. A limitation of this step was the potential of missing positive correlations (false negative) of drugs that are seldom used, or have limited entries by virtue of being newly-launched into the market. Imbalanced datasets were identified using a ratio calculated using the following formula:

$$Ratio=\frac{Number\,of\,positive\,correlated\,drugs}{Total\,number\,of\,drugs}$$

The acceptable range of dataset ratios is 0.25–0.75. Ratio of < 0.25 or > 0.75 were considered as imbalanced datasets in the current study, which was not included in any statistical analysis in model comparisons. It is expected to reach closer to the ratio of 0.4 < balance ration < 0.6 with more datasets added into the database in the future. This initial study designed for a proof of concept for the application of the database, and therefore advantageously applied the more inclusive balance ratio range of 0.25–0.75.

### Features refinement

Useful features were selected based on binary division of positive and negative datasets with significant differences on the mean of the two groups with p-value (p < 0.05) using student’s t-test. Selected features were then used for training ML models. Two types of learning datasets were built: (i) 164 average datasets through averaging data obtained from the same drug and same dose, aligning 24 × 4 = 96 features obtained from four-type of tissue tested; (ii) 4869 single datasets containing 3–10 experimental repeating datasets testing the 89 drug, > 3 doses and four types of GI tissue in different experiment preparations.

### Machine learning models

**Figure 1:**
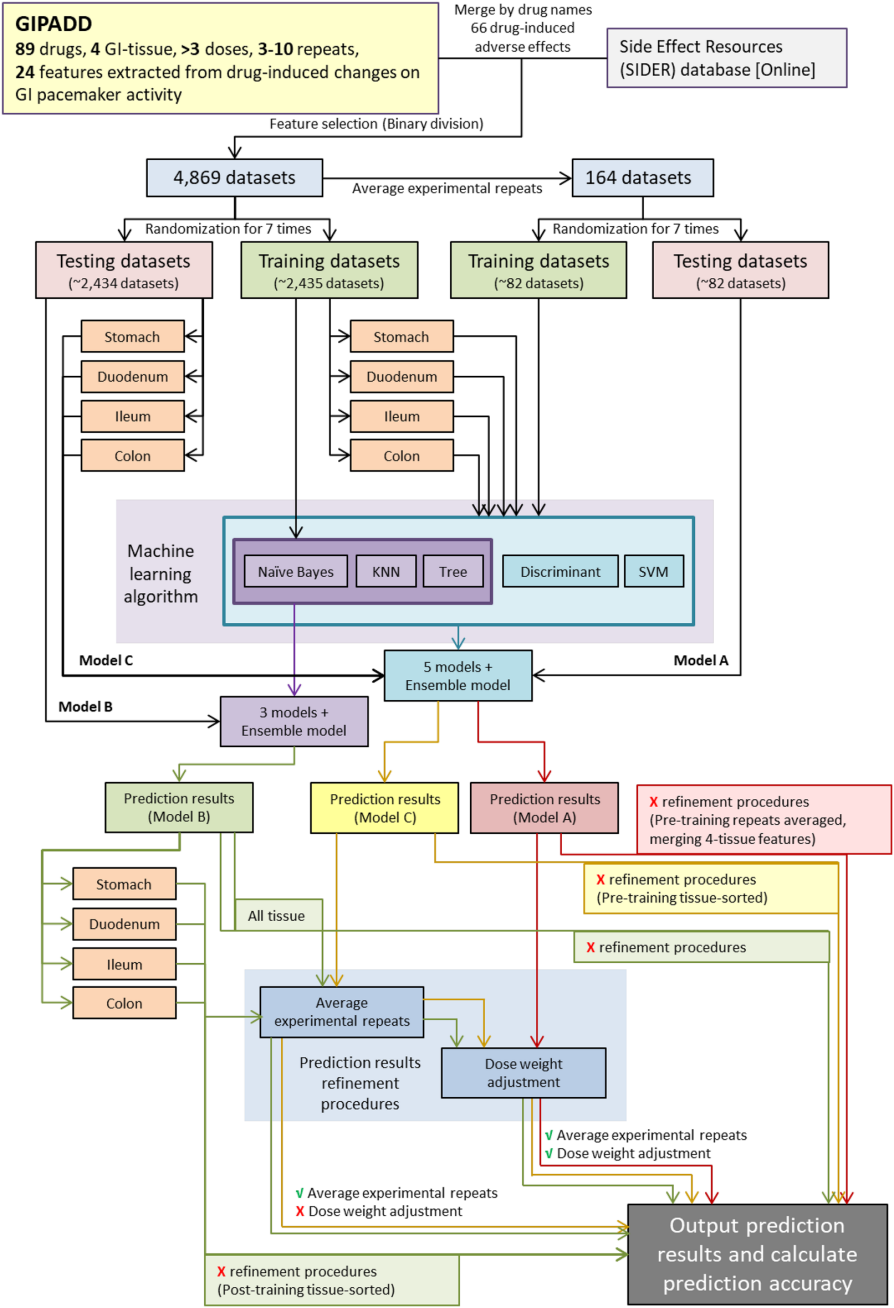
A flow chart showing the process from dataset preparation, to ML model training, to prediction result refinement for generating different types of ML models.

Several ML models were built and compared: (A) models learnt by the 164 average datasets in (i), (B) models learnt by full 4869 datasets in (ii), (C) models learnt by datasets in (ii) separated into tissue-type: stomach, duodenum, ileum and colon before training (Fig. 1). 3 types of classification algorithms were used for (B): naïve Bayes, classification tree and k-nearest neighbor (KNN) and 5 types of classification algorithms were used for (A) and (C): naïve Bayes, classification tree and KNN, discriminant analysis and support vector machine (SVM). Discriminant analysis and SVM cannot be applied in (B) because the dataset contain empty features which cannot be handled by the algorithms. An additional ensemble model was built through averaging prediction results obtained by either the three models or five models. Datasets were randomly separated into half for training and another half for testing. Randomization and training were performed for seven times to create the best model. A random dataset of the same sample size was generated based on normal distribution using mean and sample standard deviation of each feature. Models generated using random dataset were then compared with models generated using actual dataset. This step validated potential biased prediction accuracy using imbalance datasets with overfitting problems. Models which did not pass the validation test were discarded.

### Prediction results refinement

Repeated experimental datasets of the same drug and same dose in the prediction results of (b) and (c) were averaged and value > 0.5 was listed as a positive prediction result ‘1’, otherwise as a negative prediction ‘0’. Dose weight adjustment was also performed based on the assumption of higher dose may induce more severe AEs. Prediction results were adjusted by an approximately twofold weight: 1, 0.5, 0.3, 0.1 and 0.05 in descending order of dose tested (mostly separated by tenfold). The dose weight adjustment in this study is a preliminary proof of concept to test whether the tested doses of drugs may contribute to final prediction result to improve prediction accuracy, and this study describes a simple method on how it can be incorporated into the ML models to adjust final prediction results. Values of weights may be adjusted using methods like feedback Neural Network when more data is available in the future. Prediction accuracy was compared between prediction results with or without the above averaging and adjustment to deduce if these procedures may improve predictions. The use of different types of datasets and ML models for ML training, as well as post-training prediction adjustments are summarized in Fig. 1.

### Example applications of selected ML models

The selected models were tested for the application to generate adverse effect prediction report for several selected drugs. Selected models included five properties: the selected AEs for prediction, selected dataset type (a), (b) or (c) for training, algorithm-used, tissue-type, and whether or not to include prediction refinement procedures. Seven randomized predictions were performed to increase the confidence of the final prediction result. Each prediction used a randomized training datasets. Each prediction generated a result of either 0 or 1 as negative and positive correlation, respectively. The results of seven randomized predictions were averaged to create a probability value in percentage (%) representing the probability for the tested drug to induce the selected adverse effect. The number seven may be further increased when the database is larger, and an odd number is chosen to avoid tied-condition and to smoothen decision making. The AE prediction results were then compared with the SIDER database. Another three drugs which were not included in training ML model due to lack of matching drugs in SIDER were also applied for testing. Note that the current trained ML models are not calibrated due to limited datasets. Models with imbalance datasets, or failed to pass the validation test using random datasets, were not included in the final sets of AEs prediction output. Re-construction and re-training of failed models in this study is expected when the database grow larger in the further.

### Data analysis

Statistical analysis comparing different ML models was performed using PRISM 8.0 software (*GraphPad, California*). Machine learning was performed using MATLAB (*2020b; Mathworks*). All numerical data are expressed as mean ± standard deviation and p < 0.05 was considered statistically significant. Network cluster graph was plotted using R program (version 4.2.2).

## Results and discussions

### Models comparison with different pre-training and post-training refinement procedures

**Figure 2:**
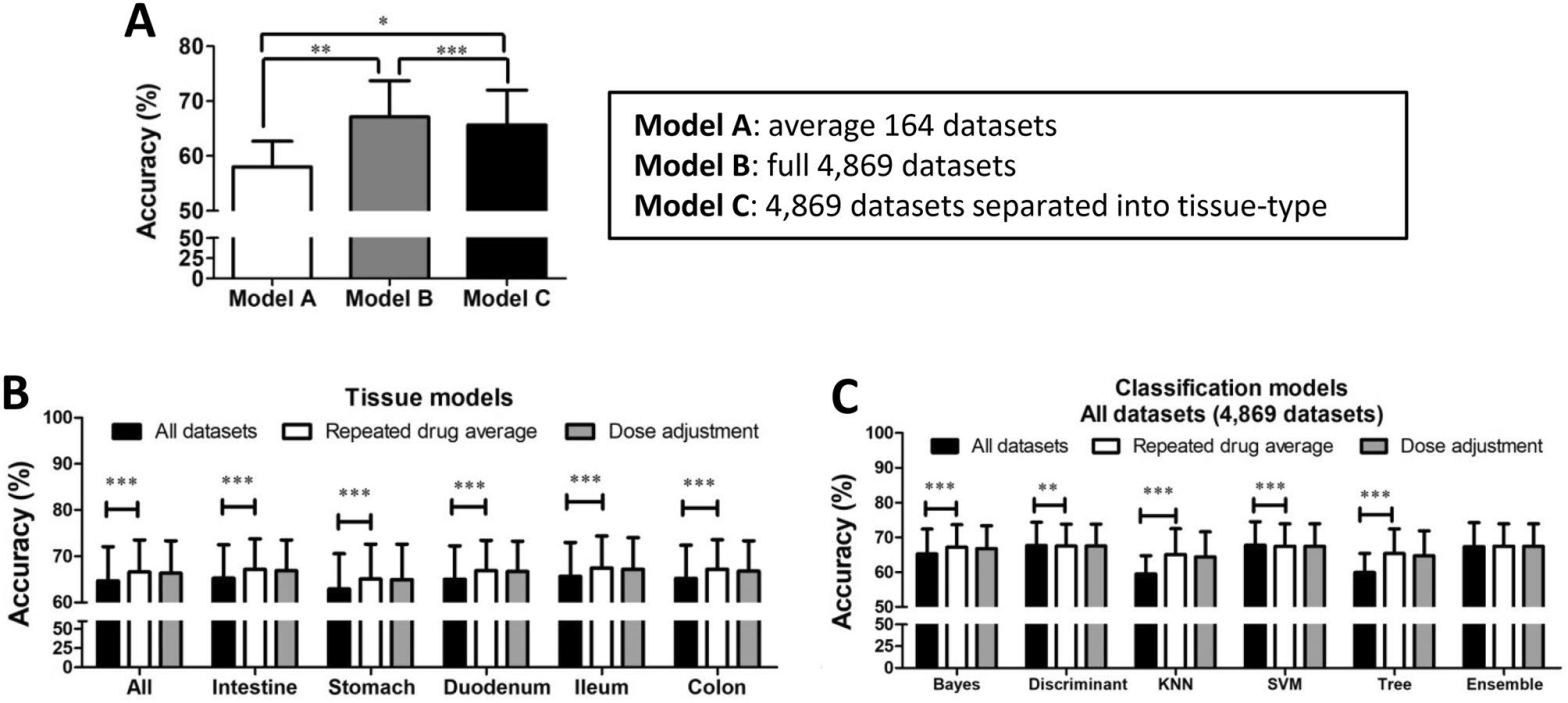
Model comparisons. **(A)** The prediction accuracy of models built using different dataset preparations for refined selected-AEs (n = 10–13); **(B)** the prediction accuracy of different tissue models. ‘All’ column indicates average of all four-tissue-type (n = 2016) and ‘intestine’ column indicates average of three intestinal segments except stomach (n = 1554), stomach and ileum (n = 462), duodenum and colon (n = 546); **(C)** the prediction accuracy of model trained using different classification algorithms (n = 336). Data represents the mean ± S.D. Significant differences are indicated as *p < 0.05, **p < 0.01, ***p < 0.001 using paired t-tests.

89 drugs were matched with the SIDER database, and the ratio of positive-correlated datasets over total number of datasets for 66 selected common AEs were calculated and listed in Supplementary Table 1. AEs with data ratio between 0.25 and 0.75 were considered acceptable, which refined 14 selected AEs. Within the 14 AEs, the average prediction accuracy was compared between different models: (A) 164 average datasets averaging experimental repeats before training (B) 4869 single datasets, (C) 4869 single datasets separated into tissue-type before training (Fig. 2A). Model B showed the best accuracy 67.1 ± 6.6% (n = 14) regardless of training algorithm and tissue-type. Model A only showed a 58.0 ± 4.7% accuracy and model C showed 65.7 ± 6.4% accuracy (n = 14). This result shows that pre-averaging experimental repeated datasets or pre-separating tissue-type before ML had generally reduced the final prediction accuracy. Between Model B and C, the difference is whether or not to isolate single tissue-type in training, and including all tissues in B improved only approximately 2% accuracy. These pre-training procedures could potentially useful for predicting certain tissue-specific AEs.

Based on Model B, the average prediction accuracy and best prediction accuracy was compared between models built using actual datasets and random datasets for the 14 AEs. 3 out of 14 AEs’ failed to identify significant features for model building using random datasets. 6 out of 11 AEs had > 0.5% better accuracy using actual datasets compared with random datasets in either average or best prediction accuracy (Supplementary Table 2). Pre-averaging training models that contain only 164 datasets suffered from limited model size between training and testing during randomization. Only models with > 80% total drugs after randomization were considered in model accuracy comparison. The prediction accuracy of the final nine selected models was 67.4–79.8%, true positive rate 62–100% and true negative rate 65–83% (Supplementary Table 3). These nine AEs ML models predict anxiety (accuracy: 79.8%), gastrointestinal disorder (79.0%), constipation (76.4%), gastrointestinal pain (75.9%), arrhythmia (74.2%), vomiting (74.1%), dizziness (70.4%), rash (70.1%) and diarrhea (69.7%). In which, anxiety is related to psychology, arrhythmia is cardiology and rash is immunology, while the rest are GI-related AEs. Arrhythmia is easy to relate, because cardiac pacemaking activity shares very similar pacemaking mechanisms with the GI^11^. For psychology-related AEs, the reason behind could be the shared receptor expression between the brain and the gut, as well as other parts of our body. For example, serotonin controls emotions^12^ and serotonin receptors are also important drug target receptors for anti-emetic therapies^13^. It is also interesting to further mention that anti-depressants are one of major group of drugs contributing to clinical unexpected GI-related AEs^14^. Rash is an immunity-related AEs, where GI contributed to 70% of our body immunity expressing many types of pre-active immune cells ready to fight against toxic substances and pathogens invasion from ingested materials^15^.

Based on these nine AEs and Model B, ML models were further compared across tissue-type (Fig. 2B) and algorithm-type (Fig. 2C). Comparisons included the refinement procedures of prediction results through averaging experimental repeated datasets and dose-weight adjustment. The result shows that the procedure of dose-weight adjustment generally did not improve the accuracy of predictions in all tested models, but rather slightly reduced the accuracy with range between 0.01 and 0.43%. The effect of dose weight adjustment may require further investigation to optimize the weight values, which may be tested again in the future with larger database. Across different tissue models, averaging repeated datasets significantly improved the average prediction accuracy for all types of tissue models by 1.8–2.2% (p < 0.001, n = 294–1428). This improvement is expected, as more experimental repeats should help improving data accuracy, provided that there is a correlation between EF and tested AEs. Across different classification algorithms, averaging repeated datasets significantly improved the accuracy only in KNN (+ 5.55%, p < 0.001, n = 238), classification tree (+ 5.45%, p < 0.001, n = 238), Naïve Bayes (+ 1.98%, p < 0.001, n = 238). However, this refinement procedure did not improved the prediction accuracy in an ensemble model (+ 0.11%, insignificant, n = 238), discriminant analysis (–0.08%, p < 0.01, n = 238) and SVM model (–0.29%, p < 0.001, n = 238). Among all tissue models, the ileum had the best accuracy at 67.4 ± 6.9%, compared to the stomach at 65.1 ± 7.6%. Across different classification algorithms, the best algorithm is discriminant analysis (67.6 ± 6.7%) and SVM (67.8 ± 6.8%) before merging experimental repeats, while KNN model showed the lowest accuracy (65.1 ± 7.4%) after merging experimental repeats. Note that the above model comparison only represents the general trend. Specific refinement procedures were sometimes found useful in improving accuracy for predicting certain AEs. The general trend found that the ileum has the best average accuracy. However, the best accuracy did not always occur at the ileum in different AEs-prediction models. For example, the model predicting vomiting has the best accuracy when using data collected from the stomach only, while model predicting gastrointestinal pain has the best accuracy using data collected from the duodenum only (Supplementary Table 3). The reasons behind could be the expression of different types and density of receptors and ion channels in different sub-segment of the gut is a better match towards that particular AE.

### Feature selection and comparison

**Figure 3:**
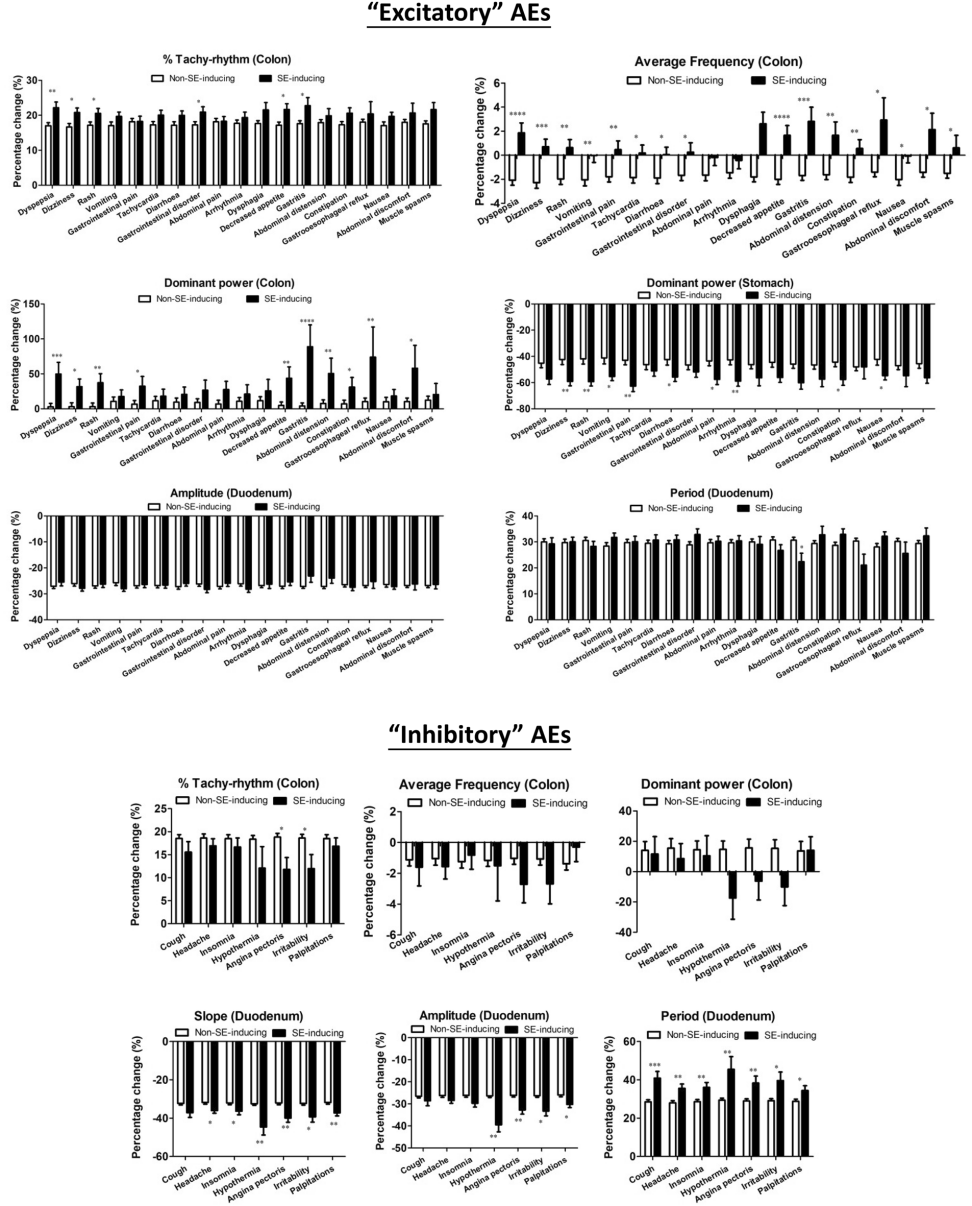
Excitatory and Inhibitory correlations to GI pacemaker activity in selected-AEs. The percentage change of selected features in various AE-inducing drugs and non-AE-inducing drugs. Data represents the mean ± S.D. Significant differences are indicated as *p < 0.05, **p < 0.01, ***p < 0.001 using unpaired t-tests (n = 46–1326).

Binary division between negative and positive AEs correlated datasets was used to select and refine features for training ML model. Although ML models may not be successfully built for all 66 selected AEs mostly due to significant imbalance in available datasets, the feature selection process could identify correlated patterns of change in EFs of GI pacemaker activity for different groups of AEs. The identified significant features could be important clues for us to correlate GI pacemaker activities to health and disease. In this study, we found that, the correlated patterns may be divided into two major groups of AEs, ‘excitatory’ and ‘inhibitory’ AEs (Supplementary Table 4, Fig. 3). AE-inducing drugs in ‘excitatory’ AEs group (19 selected AEs) had more excitatory actions on the colon, which increased average frequency, further increased tachy-rhythm power and dominant power of the colon tissues, and also further reduced dominant power of stomach tissues compare to non-AE-inducing drugs. On the other hand, AE-inducing drugs in ‘inhibitory’ AEs group (7 selected AEs) had opposite effects which did not increase average frequency, tachy-rhythm power and dominant power on the colon tissues. However, the AE-inducing drugs in the seven ‘inhibitory’ AEs shared common inhibitory effects on the duodenal tissues to further reduce the slope and amplitude, while increasing the period of waveform compared to non-AE-inducing drugs, where these changes were not identified in the ‘excitatory’ AEs. This phenomenon indicated that common patterns of change in GI pacemaker activity may be found in correlated sets of AEs. These patterns of change may be correlated to common receptor activation or inhibition to generally excite and inhibit GI pacemaker activity at different GI segments.

**Figure 4:**
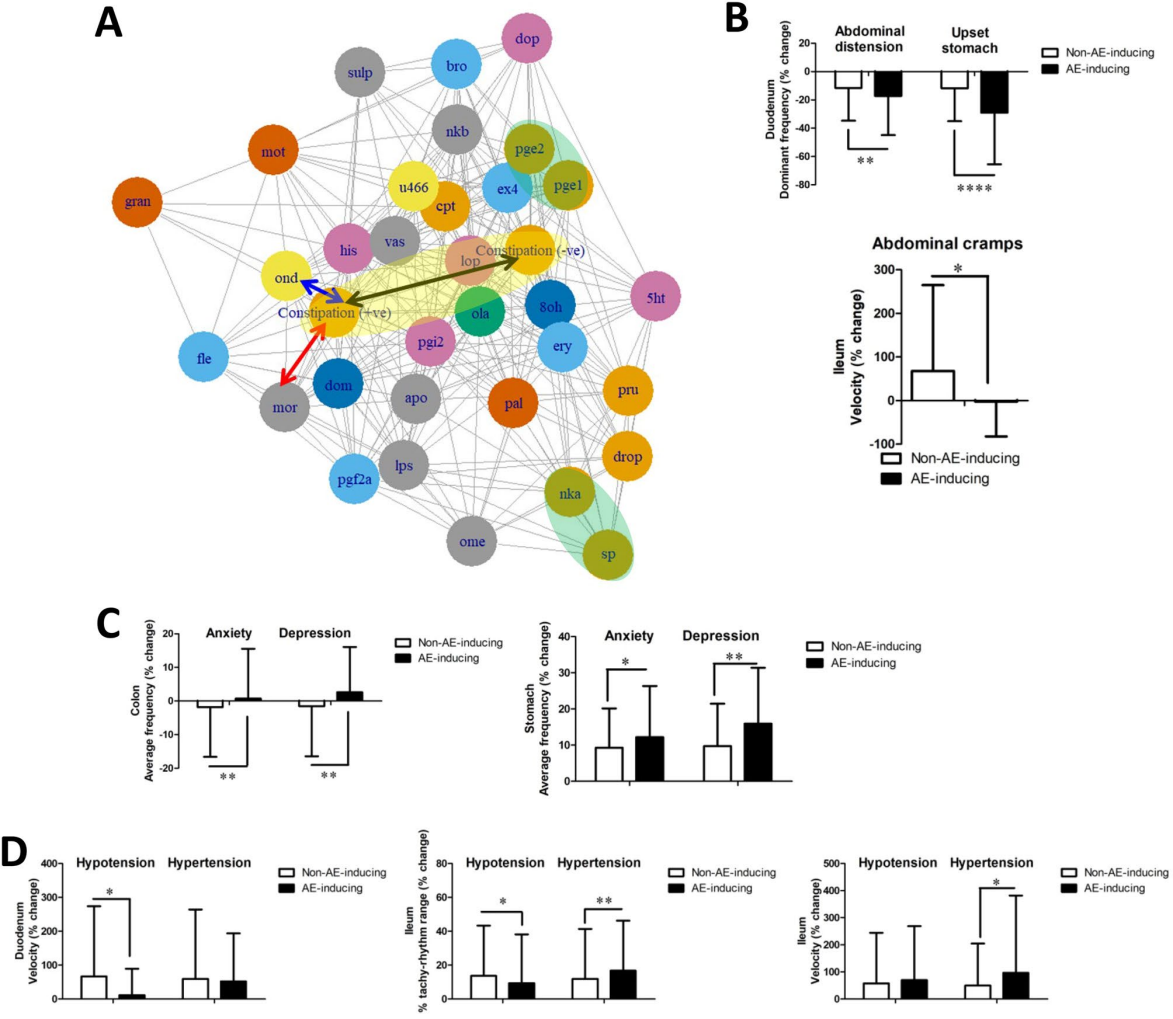
Selected GI pacemaker features correlated with AEs. **(A)** Network graph clustering selected drugs by refined EFs to an example AE: constipation. The length of black arrow represents how good the model may distinguish between positive-correlated features and negative-correlated features in constipation (shaded in yellow). Blue arrow and red arrow has a short distance to positive-correlated features of constipation, where these two drugs ondansetron (“ond”) and morphine (“mor”) are known in market to induce constipation. Drugs acting on similar receptors, such as prostaglandin E1 (“pge1”) and prostaglandin E2 (“pge2”) or substance P (“sp”) and neurokinin A (“nka”) are clustered close to each other based on EF drug profile (shaded in yellow) **(B–D)** The percentage change of selected features compared between AE-inducing drugs and non-AE-inducing drugs in **(B)** GI-related AEs,** (C)** psychology-related AEs and** (D)** hypotension vs. hypertension. Data represents the mean ± S.D. Significant differences are indicated as *p < 0.05, **p < 0.01, ***p < 0.001 using unpaired t-tests (n = 228–1300). *NKA* neurokinin A, *LPS* lipopolysaccharides.

This study also invents a graphical representation on how drugs may be correlated with AE-related EFs. Drugs may be clustered based on refined EFs and plotted into a network graph, where an example for constipation network model is shown in Fig. 4A. In which, two circles (shaded in yellow) show positive-correlation and negative-correlation with constipation respectively based on the averaged and refined supervised EFs. The larger the distance between these two circles (black arrow), the better the model may distinguish between the constipation-inducing properties of drugs. Ondansetron (blue arrow, labelled with “ond”) and morphine (red arrow, “mor”) are two drugs-in-market that are known to induce constipation as AE. Based on this graph, we can also observe that drugs known to act on similar receptors are clustered together based on EFs (shaded in green), such as prostaglandin E1 (“pge1”) and prostaglandin E2 (“pge2”), or substance P (“sp”) and neurokinin A (“nka”), providing exciting evidences that GIPADD may also potentially predict drug targeting receptors.

Other than the ‘excitatory’ and ‘inhibitory’ EF drug profiles, other interesting significant feature differences were identified. In GI-related AEs, drugs inducing abdominal distension and upset stomach had reduced the duodenal dominant pacemaker frequency at a higher level compared to non-AEs inducing drugs; and drugs inducing abdominal cramps did not alter ileum propagating velocity, but non-AE-inducing drugs generally induced it (Fig. 4B). In psychological AEs, drugs induced anxiety and depression had increased the colon average pacemaker frequency, and further induced stomach average pacemaker frequency compared to non-AEs inducing drugs (Fig. 4C). In blood pressure-related AEs, hypotension-inducing drugs reduced duodenal propagating velocity, but not hypertension-inducing drugs. On the other hand, hypotension-inducing drugs reduced percentage of tachy-rhythm at the ileum, while hypertension-inducing drugs induced it. Hypotension-inducing drugs did not change the ileal propagation velocity, but hypertension-inducing drugs induced it significantly (Fig. 4D). Although these AE-to-EF correlations maybe weak or in certain cases, very weak, these may help brainstorming novel connections and hypothesis beyond unaided human efforts. Besides, these correlations are advantageously generated based on computer calculations without the inherent bias in human-driven hypothesis.

### Drug report generation

The nine selected ML models were applied for making predictions. Four drugs, apomorphine, atorvastatin, oxytocin and amlodipine, were used for the trained models in AE prediction (supplementary Table 5). Models accurately predicted positive correlations in gastrointestinal disorder and gastrointestinal pain for amlodipine and negative correlations for apomorphine and atorvastatin. Models also predicted positive correlations in vomiting for apomorphine, constipation for amlodipine, rash for oxytocin. However, positive correlations in anxiety, arrhythmia, diarrhea and dizziness were not correctly identified for these selected drugs. In addition, another three drugs, neurokinin A (NKA), peptide YY and lipopolysaccharide (LPS), which were not used in training models due to missing side effect profile in SIDER were also tested, in which potential vomiting-inducing properties were predicted for NKA and peptide YY with 43% randomized prediction results showing positive correlations, while LPS showed very minor positive correlations with rash (14%) and constipation (14%).

## Conclusions and future studies

This report describes a selection process and prediction refinement procedures for creating the best classification ML model to predict selected AEs for drugs using the GIPADD database integrating with the SIDER database. This report also emphasize the advantages of using standardized drug screening methodology to create EF drug databases, allowing highly-consistent and massive amount of comparisons to be performed between numerous of drugs simultaneously, which could quickly advance the unexplored knowledge on drug-induced effects on EF of GI pacemaker activity, or even discover novel correlations which we had never considered before. Using our established standardized drug testing methodology with the MEA technology^1–6^ and automated data analytical pipeline^4^, any novel EF drug profile and AE prediction result may be created in 2 to 3 days. This is expected to bring game-changing impact towards decision making in drug discovery.

Addition of one new drug profile into the database allows thousands of new comparative calculations. Within the cutoff-database used in this report, there were > 6 billion comparisons and calculations made for each selected-AEs. Predictive accuracy is expected to improve with the growing GIPADD database. Its application may further extend to drug reposition, prediction of drug targets and therapeutic effects. This report only listed some of the interesting examples for the AE-to-EF correlation found using GIPADD. Using the methodology described in this report, several hypotheses were proposed, where these findings will be explored in details in our coming publications.

Lots of rooms for improvement still exist behind the AI algorithms described in this study, including calibration and refinement procedures, as well as training datasets preparation to improve model reliability. When more data are available, it may be possible to train regression model to predict the frequency of occurrence for certain AEs based on their known frequency listed in SIDER database, where these information may be more useful for end-users interpretation. This will required the continuous growth of both our database and the SIDER database to create more matched data resources. Multi-label classification models were also tested to potentially predict all listed AEs at once, but current study separate each AEs for AI model creation to rule out overfitting and data imbalance due to limited data size.

Another limitation of the current method is that some AE-of interest may not necessarily correlate to GI pacemaker activity. GIPADD focuses only on the GI pacemaker activity, while there are many other types of electrical signals producing from other tissues and organs waiting for us to translate and decode. We are currently building similar electrophysiological drug databases for pacemaker activity found in other organs, such as the heart and uterus. In the future, EF big-data may play a significant role in drug discovery and scientific development.

## Supplementary Information

Supplementary Information.

## Data Availability

The datasets used and/or analyzed during the current study available from the corresponding author on reasonable request.

## Abbreviations

GIPADD: Gastro-Intestinal Pacemaker Activity Drug Database
ML: Machine-learning
SVM: Support vector machine
KNN: K-Nearest neighbors
AEs: Adverse effects
GI: Gastrointestinal
AI: Artificial intelligence
NKA: Neurokinin A
LPS: Lipopolysaccharides
ENS: Enteric nervous system
ICC: Interstitial cells of Cajal
