# Supplementary material for: Predicting drug adverse effects using a new Gastro-Intestinal Pacemaker Activity Drug Database (GIPADD)
Source: Sci Rep. 2023 Apr 28;13:6935. doi: 10.1038/s41598-023-33655-5 (PMC10147650; doi:10.1038/s41598-023-33655-5)
Supplement: Supplementary file 1 — Supplementary Information. [file 41598_2023_33655_MOESM1_ESM.docx]

## Supplementary tables

***Supplementary-table-1*** List of selected adverse effects (AEs) analyzed in the study. AEs with acceptable ratio of [number of positive-correlated datasets/ total number of datasets] within range of 0.25-0.75 were selected for further model comparison studies.

| Adverse effects | Average accuracy  (Actual datasets) | Average accuracy  (Random datasets) | Difference |  | Best accuracy  (Actual datasets) | Best accuracy  (Random datasets) | Difference |
| --- | --- | --- | --- | --- | --- | --- | --- |
| Abdominal pain | 65.5 | 65.3 | 0.2 |  | 68.5 | 68.5 | **0.0** |
| Anxiety | 74.1 | 72.9 | **1.3** |  | 77.5 | 75.3 | **2.2** |
| Arrhythmia | 70.5 | 68.6 | **1.9** |  | 74.2 | 71.9 | **2.2** |
| Constipation | 72.1 | #1 |  |  | 76.4 |  |  |
| Diarrhoea | 62.1 | 62.0 | 0.1 |  | 69.7 | 66.3 | **3.4** |
| Dizziness | 59.7 |  |  |  | 67.4 |  |  |
| Gastrointestinal disorder | 72.7 |  |  |  | 78.1 |  |  |
| Gastrointestinal pain | 71.4 | 71.0 | 0.3 |  | 74.6 | 73.0 | **1.5** |
| Headache | 75.9 | 76.0 | **-0.1** |  | 78.7 | 79.0 | **-0.4** |
| Hypersensitivity | 68.4 | 68.3 | 0.1 |  | 71.9 | 71.9 | **0.0** |
| Nausea | 56.6 | 56.8 | **-0.2** |  | 61.4 | 62.9 | **-1.6** |
| Rash | 65.4 | 64.7 | **0.7** |  | 69.1 | 69.2 | **-0.1** |
| Tachycardia | 67.1 | 66.9 | 0.2 |  | 70.5 | 70.8 | **-0.3** |
| Vomiting | 56.4 | 57.0 | **-0.6** |  | 68.2 | 66.3 | **1.9** |

#1 No significant features were identified from the randomized datasets for model building, therefore, no prediction accuracy data were available.

***Supplementary-table-2*** Comparison between models built using actual datasets and random datasets. Table shows the average accuracy (left columns) and the best accuracy (right column) of all models created with different algorithm-type, tissue-type and with seven randomized training.

| Adverse effects | Accuracy | Algorithm | Model  Type^#1^ | Average  Repeats ^#2^ | Dose  Adjust ^#3^ | Ratio of  testing data | Tissue ^#4^ | TPR | TNR | FPR | FNR | TP | TN | FP | FN |
| --- | --- | --- | --- | --- | --- | --- | --- | --- | --- | --- | --- | --- | --- | --- | --- |
| Anxiety | 79.8 | KNN | B | Y | N | 0.75 | c | 100 | 79 | 21 | 0 | 4 | 67 | 18 | 0 |
| Arrhythmia | 74.2 | Bayes | B | Y | Y | 0.29 | a | 71 | 74 | 26 | 29 | 5 | 61 | 21 | 2 |
| Constipation | 76.4 | Tree | B | Y | Y | 0.27 | c | 76 | 80 | 24 | 20 | 20 | 4 | 64 | 20 |
| Diarrhoea | 69.7 | Tree | B | Y | Y | 0.36 | a | 62 | 72 | 28 | 38 | 13 | 49 | 19 | 8 |
| Dizziness | 70.4 | Bayes | A | N | N | 0.63 | a | 71 | 67 | 33 | 29 | 45 | 12 | 6 | 18 |
|  | 67.4 | Ensemble | B | Y | Y | 0.39 | a | 100 | 65 | 35 | 0 | 6 | 54 | 29 | 0 |
| Gastrointestinal disorder | 79.0 | Bayes | C | Y | N | 0.26 | s | 100 | 78 | 22 | 0 | 3 | 46 | 13 | 0 |
| Gastrointestinal pain | 75.9 | KNN | C | Y | N | 0.29 | d | 100 | 75 | 25 | 0 | 4 | 62 | 21 | 0 |
| Rash | 70.1 | Tree | C | Y | Y | 0.34 | d | 75 | 70 | 30 | 25 | 6 | 55 | 24 | 2 |
| Vomiting | 74.1 | Bayes | A | Y | Y | 0.69 | a | 73 | 83 | 17 | 27 | 55 | 5 | 1 | 20 |
|  | 71.0 | Tree | C | Y | N | 0.44 | s | 74 | 70 | 30 | 26 | 14 | 30 | 13 | 5 |

^#1^ **A**: 164 average datasets through averaging data obtained from the same drug and same dose, aligning 24x4 = 96 features obtained from 4-type of tissue tested; **B**: 4,869 single datasets with experimental repeated datasets testing the same drug, same dose and same tissue in different preparation for 3–10 times. **C**: 4,869 single datasets with repeating datasets and trained separately for different tissue-type.

^#2^ Prediction adjustment 1: Combine and average the prediction results of repeated datasets of the same treatment. Y: Yes; N: No.

^#3^ Prediction adjustment 2: Dose weight adjustment based on simple hypothesis that higher dose had higher chance in side effects induction by 1, 0.5, 0.3, 0.1, 0.05, currently no drugs are tested for more than 5 doses. Y: Yes; N: No.

^#4^ Representative tissue for classification: s: stomach; d: duodenum; i: ileum; c: colon; a: all.

***Supplementary-table-3*** Table showing the final selected useful ML model for predicting 9 AEs, and the properties of the selected models. **TPR**: true positive rate; **TNR**: true negative rate; **FPR**: false positive rate; **FNR**: false negative rate; **TP**: true positive count; **TN**: true negative count; **FP**: false positive count; **FN**: false negative count.

| **“Excitatory” AEs** | **“Inhibitory” AEs** |
| --- | --- |
| Common actions: excitatory actions on the colon,  reduced dominant power of the stomach | Common actions:  inhibitory effects on the duodenum |
| Dyspepsia  Dizziness  Rash  Vomiting  Gastrointestinal pain  Tachycardia  Diarrhoea  Gastrointestinal disorder  Abdominal pain  Arrhythmia  Dysphagia  Decreased appetite  Gastritis  Abdominal distension  Constipation  Gastroesophageal reflux  Nausea  Abdominal discomfort  Muscle spasms | Cough  Headache  Insomnia  Hypothermia  Angina pectoris  Irritability  Palpitations |

***Supplementary-table-4***. **List of “excitatory” AEs and “inhibitory” AEs sharing similar change of pattern based on EF drug profile.**

| Adverse effects | Drugs used in training models | | | | | | | | Drugs not included in training models | | |
| --- | --- | --- | --- | --- | --- | --- | --- | --- | --- | --- | --- |
|  | Apomorphine | | Atorvastatin | | Oxytocin | | Amlodipine | | NKA | Peptide YY | LPS |
| Anxiety | 0% | 1 | 0% | 0 | 0% | 0 | 0% | 1 | 0% | 0% | 0% |
| Arrhythmia | 0% | 0 | 0% | 0 | 0% | 1 | 0% | 1 | 0% | 0% | 0% |
| Constipation | 29% | 1 | 0% | 0 | 0% | 0 | 57% | 1 | 0% | 0% | 14% |
| Diarrhea | 0% | 1 | 0% | 0 | 0% | 0 | 0% | 1 | 0% | 0% | 0% |
| Dizziness | 0% | 1 | 14% | 0 | 0% | 0 | 0% | 1 | 0% | 0% | 0% |
| Gastrointestinal disorder | 0% | 0 | 0% | 0 | 0% | 1 | 100% | 1 | 0% | 0% | 0% |
| Gastrointestinal pain | 0% | 0 | 0% | 0 | 0% | 0 | 100% | 1 | 0% | 0% | 0% |
| Rash | 0% | 0 | 0% | 0 | 71% | 1 | 0% | 1 | 0% | 0% | 14% |
| Vomiting | 71% | 1 | 0% | 0 | 0% | 1 | 0% | 1 | 43% | 43% | 0% |

***Supplementary-table-5***. **Example drug AE prediction report.** A table showing the prediction results of 9 selected AEs for 4 selected drugs used in model training compared with AEs occurrence listed in SIDER, where ‘0’ indicates negative correlations and ‘1’ indicates positive correlations (left column), and the prediction results of another 3 drugs which was not included in SIDER and model training (right column). Red-marked values indicate wrong predictions, green-marked values indicate correct predictions, and orange-marked values indicate weak-positive predictions.

| Group | Short form of the drug name in Figure 4A | Full name of the drug |
| --- | --- | --- |
| 2 | bro | Bromocriptine |
| 3 | ola | Olanzapine |
| 4 | ond | Ondansetron |
| 4 | u466 | U46619 |
| 5 | 8oh | 8-OH-DPAT |
| 5 | dom | Domperidone |
| 6 | gran | Granisetron |
| 6 | mot | Motilin |
| 6 | pal | Palonosetron |
| 7 | 5ht | Serotonin |
| 7 | dop | Dopamine |
| 7 | his | Histamine |
| 7 | lop | Loperamide |
| 7 | pgi2 | Prostaglandin I2 |
| 8 | apo | Apomorphine |
| 8 | lps | Lipopolysaccharides |
| 8 | mor | Morphine |
| 8 | nkb | Neurokinin B |
| 8 | ome | Omeprazole |
| 8 | sulp | Sulpiride |
| 8 | vas | [Arg8]-Vasopressin |
| 9 | cpt | Cisplatin |
| 9 | drop | Droperidol |
| 9 | nka | Neurokinin A |
| 9 | pge1 | Prostaglandin E1 |
| 9 | pge2 | Prostaglandin E2 |
| 9 | pru | Prucalopride |
| 9 | sp | Substance P |
| 10 | ery | Erythromycin |
| 10 | ex4 | Exendin-4 |
| 10 | fle | Flecainide |
| 10 | pgf2a | Prostaglandin F2alpha |

***Supplementary-table-6***. **Full name of the short form of the drugs used for clustering in Figure 4A.**

| **Tissue** | **Dose** | **DF** | **AF** | **% Brady-rhythm** | **% Normal-rhythm** | **% Tachy-rhythm** | **DP** | **Amplitude** | **Slope** | **Period** | **Velocity** | **DFA(small)** | **DFA(large)** | **En(small)** | **En(large)** | **ActP** |
| --- | --- | --- | --- | --- | --- | --- | --- | --- | --- | --- | --- | --- | --- | --- | --- | --- |
| Stomach | 100uM | 8.1 | 20.3 | 7.0 | -31.5 | 24.1 | -57.0 | -18.0 | -8.2 | 3.7 | 22.0 | -4.0 | -9.8 | 2.2 | -7.9 | 19.5 |
| Duodenum | 100uM | -8.1 | -12.2 | 25.3 | -18.9 | -5.7 | -21.1 | -36.5 | -41.5 | 31.8 | 81.3 | -5.1 | -8.3 | 2.2 | -7.3 | 12.0 |
| Duodenum | 1uM | -9.7 | -7.6 | 28.0 | -26.1 | -1.6 | -34.2 | -25.3 | -31.5 | 21.0 | 97.7 | -3.3 | -7.0 | 3.2 | -1.5 | 14.6 |
| Duodenum | 10uM | -0.6 | -2.2 | 25.1 | -44.6 | 19.5 | -47.4 | -14.3 | -19.1 | 32.0 | -20.4 | -5.2 | -5.3 | 4.4 | 3.8 | 11.7 |
| Duodenum | 100nM | -7.9 | -13.1 | 51.5 | -41.3 | -10.4 | -35.0 | -41.2 | -48.3 | 30.4 | -17.6 | -3.6 | -7.1 | 0.7 | -1.8 | 11.4 |
| Ileum | 100uM | -1.1 | -0.3 | 7.3 | -20.2 | 12.6 | 12.1 | -20.8 | -25.8 | 9.8 | 33.5 | -2.9 | -8.9 | -1.6 | -11.5 | 5.9 |
| Ileum | 10uM | -8.7 | -13.5 | 30.5 | -41.7 | 11.2 | -21.6 | -30.1 | -33.6 | 32.7 | -2.0 | -4.4 | -8.2 | 1.3 | -5.3 | 8.8 |
| Ileum | 1uM | -4.7 | -3.6 | 18.8 | -25.0 | 5.8 | -51.3 | -26.3 | -31.7 | 22.5 | 65.5 | -4.7 | -7.0 | 2.9 | 2.1 | 8.4 |
| Ileum | 100nM | -11.8 | -17.1 | 28.3 | -36.6 | 9.3 | -78.0 | -28.0 | -37.5 | 39.1 | 111.1 | -3.9 | -12.2 | 1.0 | -14.1 | 13.3 |
| Colon | 100uM | -2.4 | -5.2 | 19.2 | -19.7 | 0.3 | -31.7 | -23.4 | -31.1 | 10.6 | 40.9 | -4.9 | -12.5 | 5.3 | -15.9 | 19.6 |
| Colon | 10uM | 2.6 | 5.0 | -1.2 | -29.3 | 28.8 | -25.7 | -23.7 | -23.0 | 8.8 | -10.5 | -3.2 | -7.9 | 5.2 | -8.3 | 11.3 |
| Colon | 1uM | 4.1 | 10.4 | -9.1 | -26.9 | 37.1 | 13.7 | -8.4 | -8.2 | -2.4 | 22.6 | -5.9 | -11.6 | 6.0 | -3.1 | 9.0 |
| Colon | 100nM | -2.0 | -1.8 | 9.0 | -26.1 | 17.6 | 13.9 | -5.8 | 2.9 | 13.6 | 41.7 | -5.3 | -10.0 | 2.5 | -5.6 | 16.5 |

***Supplementary-table-7*. A database fragment of an example drug, dopamine.** This data fragment is extracted from filing materials in a US provisional patent (63/268,957). We have obtained agreement for publication from the authors of this supplementary material. The 15 electrical features shown here is only part of the electrical features we used for database construction. All values represent the percentage change of some electrical features (parameters). DF: dominant frequency; AF: average frequency; DP: dominant power; ActP: change in activation pattern. %Brady-rhythm, %Normal-rhythm, %Tachy-rhythm: parameters extracted using power spectrum segmentation. DFA(small) and DFA(large): parameters extracted using detrended fluctuation analysis. En(small) and En(large): parameters extracted using sample entropy algorithm. Only brief description of the material is provided here. For further technical explanatory details, please refer to the patent’s supplementary notes.

**
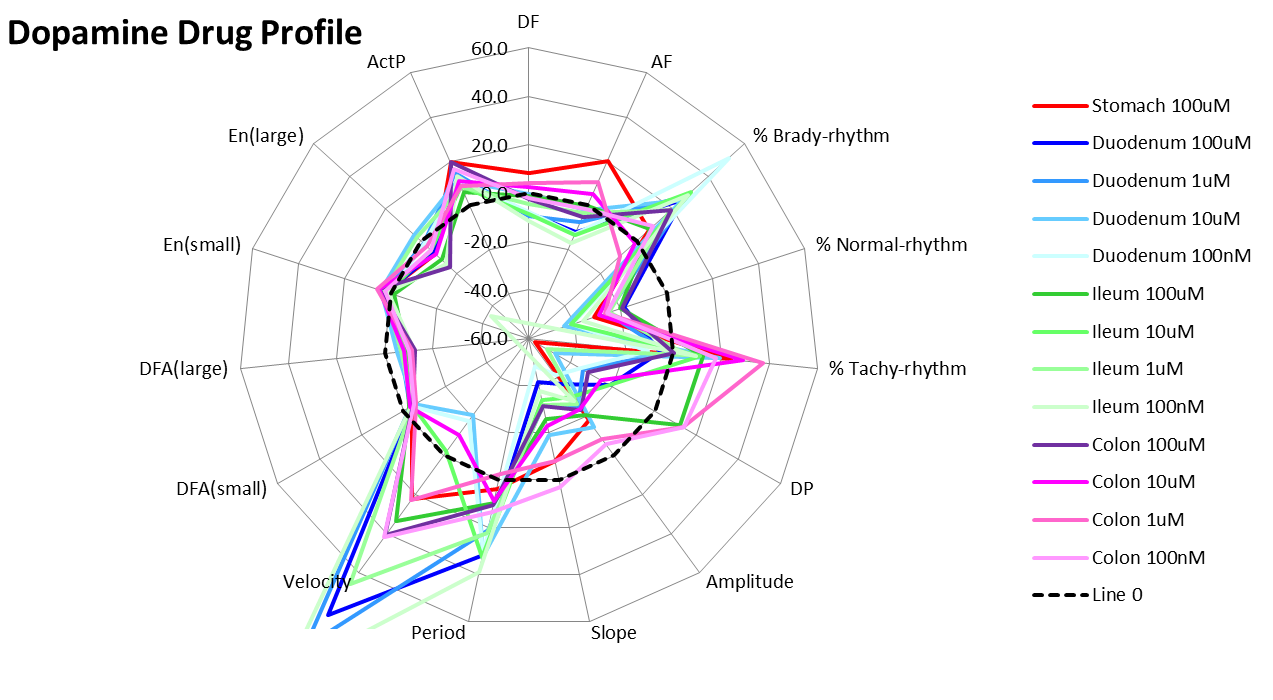
**

***Supplementary-figure-1*. A radar diagram showing an electrical drug profile of dopamine.** Data are extracted from supplementary table 7. This data fragment is extracted from filing materials in a US provisional patent (63/268,957). We have obtained agreement for publication from the authors of this supplementary material.
